# The need for consensus on delineation and dose constraints of dentofacial structures in paediatric radiotherapy: Outcomes of a SIOP Europe survey

**DOI:** 10.1016/j.ctro.2023.100681

**Published:** 2023-09-24

**Authors:** Angela Davey, Shermaine Pan, Abigail Bryce-Atkinson, Henry Mandeville, Geert O. Janssens, Sarah M. Kelly, Marinka Hol, Vivian Tang, Lucy Siew Chen Davies, SIOP-Europe Radiation Oncology Working Group, Marianne Aznar

**Affiliations:** a Division of Cancer Sciences, Faculty of Biology, Medicine and Health, The University of Manchester, Manchester, UK; b Department of Proton Therapy, The Christie NHS Foundation Trust, Manchester, UK; c The Royal Marsden NHS Foundation Trust, London, UK; d Princess Maxima Center for Paediatric Oncology, Utrecht, The Netherlands; e Department of Radiation Oncology, University Medical Center Utrecht, Utrecht, The Netherlands; f European Society for Paediatric Oncology (SIOP Europe), Clos Chapelle-aux-Champs 30, Brussels, Belgium; g The European Organisation for Research and Treatment of Cancer (EORTC) Headquarters, Avenue E. Mounier 83, Brussels, Belgium; h Faculty of Medicine and Health Sciences, Ghent University, Ghent, Belgium; i Department of Otorhinolaryngology, University Medical Center Utrecht, Utrecht, The Netherlands; j Paediatric Radiology, Royal Manchester Children’s Hospital, Manchester, UK; k Department of Radiotherapy, The Christie NHS Foundation Trust, Manchester, UK

**Keywords:** Dentofacial, Radiotherapy, Paediatrics, Late adverse effects, Contouring, Dose-volume constraints

## Abstract

**Background and purpose:**

Children receiving radiotherapy for head-and-neck tumours often experience severe dentofacial side effects. Despite this, recommendations for contouring and dose constraints to dentofacial structures are lacking in clinical practice. We report on a survey aiming to understand current practice in contouring and dose assessment to dentofacial structures.

**Methods:**

A digital survey was distributed to European Society for Paediatric Oncology members of the Radiation Oncology Working Group, and member-affiliated centres in Europe, Australia, and New Zealand. The questions focused on clinical practice and aimed to establish areas for future development.

**Results:**

Results from 52 paediatric radiotherapy centres across 27 countries are reported. Only 29/52 centres routinely delineated some dentofacial structures, with the most common being the mandible (25 centres), temporo-mandibular joint (22), dentition (13), orbit (10) and maxillary bone (eight). For most bones contoured, an *‘As Low As Reasonably Achievable’* dose objective was implemented. Only four centres reported age-adapted dose constraints.

The largest barrier to clinical implementation of dose constraints was firstly, the lack of contouring guidance (49/52, 94%) and secondly, that delineation is time-consuming (33/52, 63%). Most respondents who routinely contour dentofacial structures (25/27, 90%) agreed a contouring atlas would aid delineation.

**Conclusion:**

Routine delineation of dentofacial structures is infrequent in paediatric radiotherapy. Based on survey findings, we aim to 1) define a consensus-contouring atlas for dentofacial structures, 2) develop auto-contouring solutions for dentofacial structures to aid clinical implementation, and 3) carry out treatment planning studies to investigate the importance of delineation of these structures for planning optimisation.

## Highlights

• 52 paediatric radiotherapy centres responded to a dentofacial structures radiotherapy practice survey.
• Just over half of paediatric centres delineate at least one dentofacial structure in routine practice.
• The main barrier to delineation of dentofacial structures is lack of contouring guidance.
• Most centres (90%) agree a contouring atlas would aid delineation of dentofacial structures.
• Few centres implement specific dose objectives for dentofacial structures.

## Introduction

Worldwide, approximately 400,000 children and adolescents are diagnosed with cancer each year [1]. Tumours located in the brain, central nervous system (CNS), intracranial, or head-and-neck account for over a third of such diagnoses [2], [3]. Due to significant advancements in cancer treatment over the past 40 years, up to 85 % of children living with cancer in high-income countries will survive five years or more, and many will be cured [4], [5]. Radiotherapy plays a main role in curative treatment [6], [7], but due to increasing survival after irradiation, childhood cancer survivors are at significantly increased risk of developing late toxicities [8].

Children with head-and-neck tumours are at risk of experiencing severe late adverse effects that affect the face and teeth, due to dose received by the dentofacial structures. In particular, children irradiated before their pubertal growth spurt experience dentofacial side effects due to the radiation having a growth halting effect on the development of bones and tissues [9]. Impacted dentofacial structures will include (but are not limited to) the mandible, temporo-mandibular joint (TMJ), nasal bones, ethmoid bones, sphenoid, maxillary bones, dentition, and orbit. As a child ages and un-impacted bones grow, facial disfigurement, hypoplasia or clinical asymmetry will become more apparent. Clinically significant facial asymmetry has been reported in ∼ 77 % of children treated with radiotherapy to the head-and-neck [10]. Dental problems in this population such as tooth absence, microdontia, or defective roots/enamel have also been reported at rates between 33 % and 100 % [9], [11], [12]. The variation in rates is likely a result of the heterogeneity of dose to whole bones, or bony sub-structures, which results in areas of high, intermediate and low dose, all with different effects on bony development [11]. Other dental problems such as caries can also occur secondary to salivary dysfunction due to dose received to the salivary glands [12], [13].

Despite the high incidence of dentofacial side effects in childhood cancer survivors – dose to dentofacial structures is infrequently reported compared to dose–response relationships for other late adverse effects such as, chronic endocrine or neurologic conditions [14]. However, dentofacial side effects are *life-altering* and are linked to emotional distress and reduced quality of life [15]. Dysfunction can also occur secondary to these effects, such as difficulties in respiration and alimentation [14]. Further to this, reconstructive options are very limited, therefore prevention of dentofacial side effects would be preferable.

So far, understanding of dose–response relationships for dentofacial structures is limited. In a review from the Paediatric Normal Tissue Effects in the Clinical (PENTEC) on dental and salivary side effects, a consensus recommendation of mean dose to the teeth of less than 20 Gy for children less than 4 years old was reported based on limited evidence [12]. There is yet to be a consensus reported for facial bones, with a limited sample of studies defining dose constraints from retrospective data (e.g., maximum dose < 40GyRBE to the orbital rim [16]). However, we expect such dose constraints will depend on the individual facial bone and age of the patient, as bones develop at different rates in a growing child. For example, most of the calvarium is usually developed by the age of 5, but craniofacial growth centres continue developing in the second decade of life [17]. With increasingly more conformal treatments being implemented clinically, including proton beam therapy, more specific sub-structures (or growth centres/sutures) for dose reporting may be required.

A potential challenge in the implementation of dose constraints to dentofacial structures is the contouring of these structures on radiotherapy planning computed tomography (CT). There is currently no literature reporting on contouring practices for dental or facial structures in paediatrics [18]. In this article, we report on a survey that aims to gain increased understanding of current practice in dose assessment and contouring of dentofacial structures in paediatric radiation oncology centres. We also investigate potential future initiatives to work towards minimising dentofacial side effects in childhood cancer survivors.

## Methods

A digital survey was distributed via email to European Society for Paediatric Oncology (SIOP Europe) members of the Radiation Oncology Working Group (ROWG), and member-affiliated centres in Europe (see: https://siope.eu/Radiation-Oncology-Centres), Australia, and New Zealand (a preview of the survey is available as Supplementary Material A). On distribution, we requested that each paediatric radiation oncology centre would submit only one response. Centres were asked to confirm consent to participate in the questionnaire, and progression through all sections of the online survey was automatically recorded by Qualtrics software, QualtricsXM, Copyright © 2022 [19]. Centres were given one month to respond before the survey was closed.

All responses were collated for analysis and visualisation using R version 4.0.2 [20]. Responses were excluded if consent was not confirmed, or institution name was missing. For centres with multiple responses, we included the entry that was most complete (i.e., response with the fewest number of skipped questions). If the same number of questions were complete in both responses, one entry was selected at random.

The questionnaire consisted of two sections. **Section A** (question (Q)1 – Q5) collected data on the current clinical practice with delineation of dentofacial structures and barriers to assessing dose to dentofacial structures in clinical practice. The complete list of questions is included as Supplementary Material. The survey questions were developed by a multi-disciplinary team (paediatric consultant oncologist, maxillofacial surgeon, expert in late-effects and a medical physics researcher) and adapted in discussion with SIOPE ROWG. Additionally, a wide literature search was performed to identify the potential structures of interest. All questions were based on challenges radiation oncologists involved were currently facing in routine clinical practice. Centres that reported contouring at least one dentofacial structure in routine practice (Q1) were asked to provide details on contouring practice and radiotherapy dose tolerances used in Q6. **Section B** focused on potential areas for future development (Q7) and participant information for further communication (Q8 and Q9). Any free-text responses to the survey were encoded by theme by the first author in discussion with the other co-authors.

## Results

### Response collection

**Fig. 1:**
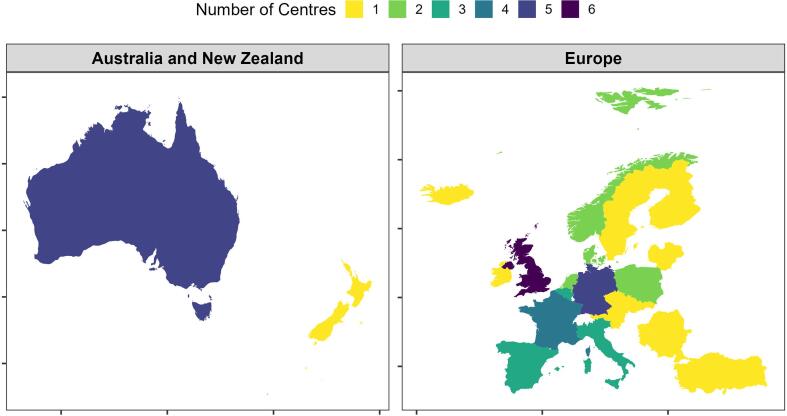
Locations of the centres who participated at the survey represented by a frequency map of the number of centres per country. Colours on the map scale from one to six centres per country.

The survey was distributed to 410 individual contacts, which was expected to cover approximately 121 listed institutions across 31 different countries. In total, 60 responses were complete at survey closure. After excluding four duplicate entries and four responses with missing department information, 52 complete responses from 27 countries were available for analysis (46 Europe, five Australia, and one New Zealand). The final response rate was 43 % (52/121) of listed institutions, covering 87 % (27/31) of countries that were included. The distribution of those 52 centres per country is shown in Fig. 1.

### Current practice

**Fig. 2:**
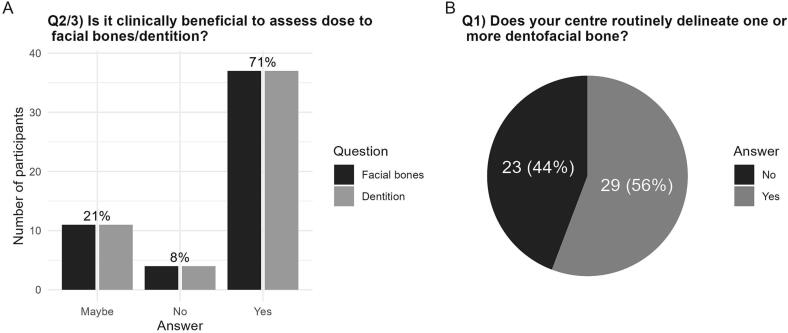
**A)** A bar chart representing the benefit of assessing the dose to facial bones and dentition in routine practice. Results are presented for facial bones (dark grey) and dentition (medium grey). **B)** Percentage of departments routinely contouring one or more dentofacial structures in routine practice.

Most centres reported that dose assessment of dentofacial structures is clinically beneficial in children yet to reach their pubertal growth spurt 37/52 (71 %), and 11/52 (21 %) were unsure. The four respondents who did not find it clinically beneficial cited reasons related to lack of knowledge on dose–response (2 answers) and questioned the feasibility of implementation (3 answers). Similar responses were recorded for dentition (Fig. 2A).

**Fig. 3:**
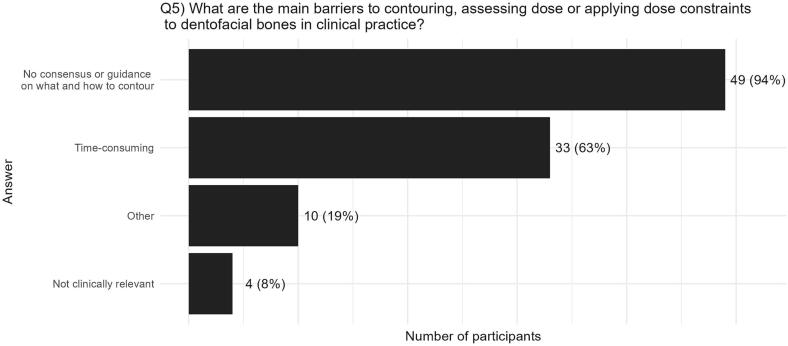
A bar chart representing the consensus across centres on the main barriers to contouring, assessing dose, or applying dose constraints to dentition or facial bones in routine clinical practice.

Despite the interest in assessing dose to dentofacial structures, only 29 centres (56 %) are routinely delineating one or more dentofacial structures in practice (Fig. 2B). The main barriers to dose-assessment were primarily a lack of guidance on which, and how, anatomical structures should be delineated (49/52, 94 %) and secondly, time required to delineate (33, 68 %) (Fig. 3). Ten out of 52 centres (19 %) provided a free-text response to this question, with the majority suggesting research is required to determine clinical relevance and assess the impact of conformal radiotherapy practices (e.g., intensity modulated radiotherapy (IMRT) (5/10 answers)).

In relation to the potential salivary impact on dentition, 21/52 centres (40 %) did not consider the minor salivary glands, 27/52 (52 %) included them in a composite oral cavity contour, and three centres delineated them separately.

### Contouring and dose assessment

**Fig. 4:**
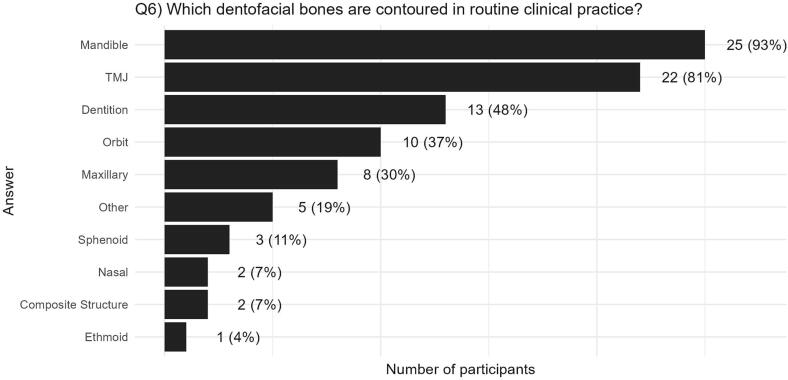
Number (and percentage) of centres that contour each bone in clinical practice (out of 27 centres that completed this information).

Out of the 29 centres who contour one dentofacial structure as a minimum, 27 centres answered questions relating to their practices. The results in this section therefore report on the 27 centres who provided this information. Fig. 4 displays the distribution of individual structures contoured by these centres. Two centres contoured a *‘composite structure’* rather than specific dentofacial structures; one of these centres stated that the composite structure encompasses the mandible, maxilla, nose, and oral cavity. Four centres reported additional soft-tissue structures that are contoured, which were the oral cavity, salivary glands, parotid glands, and pharyngeal constrictor muscles.

The centres who completed this section provided contouring and dosimetric information for each dentofacial structure individually. Most centres reported that Radiation Oncologists (RO) complete all contouring (13/27), while 3/27 reported that a Radiographer or Dosimetrist (RTT) led contouring, and 5/27 divided the responsibility between ROs and RTTs. Two centres implemented automated tools but provided limited information on specific product names and versions. Four centres did not provide any contouring information for the specific structures.

Although most centres did not report using an automated solution in current practice, 19/29 centres (66 %) were interested in having an automated solution as an option for aiding the delineation of dentofacial structures. Only 19/27 (70.4 %) centres stated they were confident in reviewing contours for dentofacial structures and 26/27 agreed that an atlas would aid delineation and/or review of auto-contours.

Dose objectives implemented in clinical practice for each structure are displayed in Table 1, for most centres this was an “as low as reasonably achievable” (ALARA) objective. A small number of centres (8/27) defined a mean (Dmean) or maximum dose constraint (Dmax) constraint for a specific structure. Age adapted dose constraints were used in 4/27 centres. For this small number of centres, dose constraints were determined by puberty or facial development (i.e., if growth-plates are fused) but no information was provided on how this is assessed in practice.

**Table 1:** Details of dose objectives reported in the survey. The completion column shows the number of centres that provided dosimetric information for each structure, as a percentage of the number of centres that were included in the overall survey results. The table reports the number of these who use an ALARA dose objective or a specified dose constraint.

	Completion Rate (number of centres reporting dose objective/number of centres overall)	Dose objective				DMax Range [Gy] (count)	DMean Range [Gy] (count)
		ALARA	Specified constraint	Both	Unknown		
**Mandible**	23/52 (44 %)	17	3	2	1	60 – 72 (n = 5)	20 – 60 (n = 2)
**TMJ**	20/52 (38 %)	15	1	3	2	50 – 60 (n = 3)	20 – 30 (n = 2)
**Dentition**	12/52 (23 %)	8	1	2	1	10 – 20 (n = 2)	10 (n = 1)
**Orbit**	8/52 (15 %)	6	0	1	1	–	20 (n = 1)
**Maxillary**	7/52 (13 %)	4	1	1	1	–	20 (n = 1)
**Sphenoid**	3/52 (5.8 %)	2	0	0	1	–	20 (n = 1)
**Nasal**	2/52 (3.8 %)	1	0	0	1	–	20 (n = 1)
**Ethmoid**	1/52 (1.9 %)	1	0	0	0	–	–

### Future initiatives

Most centres (49/52, 94 %) were interested in initiatives to improve understanding of dentofacial late adverse effects post-radiotherapy for childhood cancer. In particular: 88 % of centres (46/52) were willing to participate in an online contouring workshop, 63 % (33/52) expressed interest in an online symposium to discuss current research and the potential for future collaboration, and 28/52 centres were willing to join a working group.

## Discussion

While it is known by the radiotherapy community that it is important to evaluate dose to dentofacial structures, we demonstrate with quantifiable numbers how infrequently this is done in clinical practice. For example, only 56 % of centres across Europe, Australia and New Zealand currently delineate one or more dentofacial structures as standard. We have also identified lack of contouring guidance for dentofacial structures as the largest barrier to clinical implementation. This survey raises awareness of the lack of evidence in this space and motivates the clinical need for a solution.

As far as the authors are aware, this is the first survey aiming to understand current international practice in contouring and dose assessment to dentofacial structures in radiotherapy for children with head-and-neck tumours. Although clinical practices have not been directly reported on, the link between higher radiation dose, younger age, and dentofacial toxicities is documented [14]. Only eight centres reported on specific dose objectives to few structures (i.e., mandible, temporo-mandibular joint, dentition). Perhaps not surprisingly, we did not find a formal systematic review on dose–response evidence for dentofacial effects in head-and-neck radiotherapy for children. The closest to this was evidence collated by PENTEC on dental and salivary dose–response, [12], but this has not been extended to include facial structures. This has motivated the need for such work, and we are currently performing this systematic review.

The availability of current evidence is important when considering the time burden of delineation – as 63 % of paediatric centres highlighted contouring time as a significant barrier. In the survey, we did not ask centres to report the time taken to delineate specific dentofacial structures, as we felt this would be sensitive to the expertise and training of the individual contouring. From our own experience, we estimate this could be anywhere between 10 and 15 min for the mandible, to over 3 h for the complete set of structures included in this survey. If we combine this with contouring time for other organs at risk, and the clinical target volume – this would be greater than 4 h for a single case [21], [22]. Considering this in the context of current clinical trials, the rhabdomyosarcoma trial, FaR-RMS (NCT04625907), emphasises the importance of contouring the facial bones, but guidelines are not yet available to aid the delineation process. This further reflects the importance of raising awareness on the challenges faced when assessing dose–response in routine practice.

To identify dose–response relationships, there is a need for accurate, objective measurement of toxicities to enable large-scale retrospective studies. Such studies could lead to implementation of specific dose constraints that will provide evidence of reduction of dentofacial side effects in clinical practice. On developing such dose constraints, it is imperative to consider other factors such as age, gender, tumour location, and previous surgery. In this survey, we found that age-adapted dose constraints are infrequently implemented clinically (4/52 centres), despite the fact that age at the time of treatment is known to impact the risk of dental abnormalities [12], and facial disfigurement [10]. In fact, most (but not all) facial asymmetry has been observed in those patients treated before the pubertal growth spurt [23]. However, we expect this will depend on tumour location as different facial bones develop at differing rates [17].

As a starting point to develop dose–response evidence, existing data repositories could be utilised. The recommendation to keep mean dose to dentition < 20 Gy for children less than 4 years old, where possible, stated by PENTEC was based primarily on analysis of the Childhood Cancer Survivor Study (CCSS). This analysis identified that mean dose to the jaw of < 20 Gy was associated with increased risk of dental abnormalities in children under the age of ten at irradiation, and doses greater than 20 Gy increased the risk for all ages [16]. Older reports were also included in this review, which suggested doses as low as 4 Gy to the developing dentition is linked to tooth abnormality [17]. But, the data-sets available were limited, and only one study reported detailed dose–response data on individual teeth [13]. For facial structures, retrospective data-sets have been utilised to derive dose–response relationships for the orbital region [16]. A limitation in many of these analyses is that treatment techniques are typically historic (which is partially influenced by the long-term follow-up required for study) [14]. To support this, effort should be made to capture and evaluate delineation and late adverse effect data in routine clinical practice as well as new prospective trials. A SIOPE project coined QUARTET (Quality and Excellence in Radiotherapy and Imaging for Children and Adolescents with Cancer across Europe in Clinical Trials) supports the radiotherapy quality assurance for prospective trials and will help to capture such data for future evaluation. The reporting of late adverse events is included in some QUARTET affiliated rhabdomyosarcoma trials such as FaR-RMS (NCT04625907) from the European paediatric soft tissue sarcoma group (EpSSG), but further long term follow up studies are required to support this effort.

Despite limited dose–response evidence, 92 % of centres felt that there is a clinical benefit to delineating and assessing dose to dentofacial structures in routine practice. An important point to consider is that a better understanding of dose–response may not always lead to plan adaptation to allow better sparing and balance of planning objectives than previously possible. However, the reduction of the total dose to organs-at-risk may be more likely with conformal radiotherapy techniques (IMRT and proton beam therapy) e.g., by adjusting the number of beams or the angle of beam entry [24]. It will also be imperative to analyse freedom we have for adjustment in different clinical cases (e.g., parameningeal vs orbital rhabdomyosarcoma). In addition, a greater understanding of the dose response relationship and additional information on the risk can better inform patients and guardians on potential toxicities during decision making for treatment preferences as well as long-term follow-up practices [25]. This aids the process of gaining informed consent from parents before the initiation of radiotherapy.

In this study, we identified some limitations in our survey design. Firstly, the answers rely on the knowledge and experience of the respondent who is completing the survey on behalf of the centre, and we did not request this information. Secondly, some of questions rely on the respondent’s subjective interpretation based on individual clinical experience, i.e., Q2/3 asked respondents if they think assessing dose to dentofacial structures is clinically beneficial, and Q6f asked how confident respondents felt in reviewing or editing auto-contours. Although we hope responses to such questions were discussed within each individual centre, we cannot confirm that this was the case. Furthermore, each response could be limited by recall-bias. In a survey requesting contouring information, it is unlikely that details of contouring that is performed will be omitted. It is more likely that respondents will overestimate the contouring performed in practice. As a result, correction for recall-bias would likely provide further support to our conclusions.

Finally, as there are limited dose constraints implemented in current practice, there is a limited sample of centres (29/52, 56 %) who report on dosimetric and contouring information on facial bones. Furthermore, as only four centres (8 %) implement age-adapted dose constraints, we could only make limited conclusions on responses provided by a small number of centres. Additionally, only a few centres implemented auto-contouring solutions, and there was limited free-text information on product details and specific versions. These centres reported on a mix of commercially available and in-house manufactured research solutions, including: syngio.via RT Image Suite (Siemens), Raystation auto-contouring atlases (RaySearch Laboratories AB, Sweden), tools from Mirada Medical Limited (Oxford, UK) and a homemade tool (*Dosimetric Evaluation of Risk of Osteoradionecrosis: DERO*) [26]. As far as the authors are aware, no literature has been reported on commercial solutions for contouring specific dentofacial structures in paediatric patients – so we cannot conclude whether such solutions are feasible to implement or beneficial for paediatrics.

Future development of automated tools could aid delineation of dentofacial bones specific to paediatric anatomy [27]. Most treatment planning systems (TPS) will accept DICOM-RT Structures from outside of the TPS. Automated tools could be designed as a third-party solutions (e.g., atlas or deep-learning based [28]) with DICOM-RT output, so that the automatically contoured structures could be imported into any TPS. Alternatively (and arguably preferably in terms of workflow) automated tools would be incorporated into many TPS’s, but this would require collaboration with vendors and a commitment on their part to provide dedicated solutions for this unique, but small, patient group where commercial incentive may be limited. Alongside the development of such tools, retrospective analysis techniques, such as, *image-based data mining* could help identify particular (sub-)structures of importance [29], [30]. In this survey, the mandible, TMJ, and dentition were the most common delineated bones across paediatric radiotherapy centres – likely reflective of the fact that there are guidance and contouring atlases available for these bones. However, such atlases are primarily developed for adult head-and-neck radiotherapy [31], [32], [33], and it is unknown whether these translate to developing bones and teeth in children [31]. Overall, contouring atlases are widely used in radiation oncology to standardise volumes, harmonise contouring across centres, reduce inter-observer variability and reduce the time taken to contour [34]. In our survey, we found 90 % of centres contouring dentofacial bones routinely would be interested in a contouring atlas to aid delineation. This would require development of an atlas for dentofacial bones specific to paediatrics.

Despite awareness that most paediatric patients treated with radiotherapy to the head-and-neck will experience dentofacial side effects after treatment, there is limited delineation and implementation of dose constraints in clinical practice. This survey identified that the biggest barrier to dose assessment is the lack of consensus on contouring guidelines and the time-consuming process of delineating dentofacial structures routinely. Based on the findings of this survey we propose to implement three initial steps: 1) define a consensus-contouring atlas for dentofacial structures, 2) develop auto-contouring solutions based on the atlas to facilitate implementation in clinical practice, and 3) clarify the possibility of reducing dose to dentofacial structures without compromising tumour coverage by carrying out treatment planning studies for both photon and proton beam therapy. The above steps are in line with the commitment of QUARTET, SIOPE ROWG and the EpSSG to support the development of additional tools within clinical trials and radiotherapy guidelines. In addition, the aforementioned steps will facilitate multi-centre studies that aim to identify age-adapted dose constraints in this population. Once dose constraints (or new sub-structures at risk) are established, treatment planning studies can be focused on new areas and auto-contouring solutions can be updated to follow new guidelines. To ensure we can combat barriers to implementation of contouring guidelines and dose constraints we have started a multi-centre dentofacial working group named SMILE *(‘minimiSing long-terM Impact on dentition and faciaL asymmEtry in childhood cancer survivors’*) and are working closely in collaboration with existing working groups and projects such as SIOPE ROWG and QUARTET.

## CRediT authorship contribution statement

**Angela Davey:** Writing – original draft, Writing – review & editing, Visualization, Methodology, Data curation, Investigation, Conceptualization. **Shermaine Pan:** Supervision, Conceptualization, Writing – review & editing, Methodology. **Abigail Bryce-Atkinson:** Writing – review & editing, Methodology. **Henry Mandeville:** Writing – review & editing, Methodology. **Geert O. Janssens:** Writing – review & editing, Methodology. **Sarah M. Kelly:** Writing – review & editing. **Marinka Hol:** Writing – review & editing, Conceptualization. **Vivian Tang:** Writing – review & editing. **Lucy Siew Chen Davies:** Writing – review & editing. **SIOP-Europe Radiation Oncology Working Group:** Project administration, Resources. **Marianne Aznar:** Writing – review & editing, Supervision, Conceptualization, Methodology.

## Declaration of Competing Interest

The authors declare that they have no known competing financial interests or personal relationships that could have appeared to influence the work reported in this paper.
