# Supplementary material for: The need for consensus on delineation and dose constraints of dentofacial structures in paediatric radiotherapy: Outcomes of a SIOP Europe survey
Source: Clin Transl Radiat Oncol. 2023 Sep 24;43:100681. doi: 10.1016/j.ctro.2023.100681 (PMC10543782; doi:10.1016/j.ctro.2023.100681)
Supplement: Supplementary Data 1 [file mmc1.docx]

Current clinical practice and future development in contouring of dental or facial bones.

Consent form and information

**Q0**
Thank you for taking the time to participate in this survey about **radiotherapy and dental or facial bones**. We would like to invite each radiation oncology department to **submit one response**. 
 
Living with dental or facial effects post-radiotherapy can be challenging for our patients. The delineation of dental or facial bones is not part of routine clinical practice and is an area with very little international recommendation. We would therefore value information from **all** radiation oncology departments of its current practices.
 
This two-part survey aims to:

**Section A (Questions 1 - 6):**evaluate the current clinical practice with delineation of **facial bones and dentition** (including use of auto-contouring), radiotherapy dose tolerances used, and barriers in clinical practice.

**Section B (Questions 7 - 9):** establish areas for future development and interest in dentofacial effects post-radiotherapy.

This survey will take less than ten minutes to complete. 
 
If you have any questions relating to this survey, please direct your queries to Dr Shermaine Pan (s.pan@nhs.net).
 
Thank you once again for your contribution.

I consent and want to continue with the survey

I do not consent and do not want to participate in this survey

**Section A Current Practice**

**Q1**

The contouring of **dental or facial bones** on radiotherapy planning CT for paediatric patients is not routinely practised, and this survey hopes to better understand the current practices in radiation oncology departments in Europe. 

 
The term **facial bones** includes (but is not limited to) the mandible, temporomandibular joint (TMJ), nasal bones, ethmoid bones, sphenoid bones, maxillary bones, and orbit. The term **dentofacial bones** also includes dentition. 
 
Does your department routinely delineate **one or more** dentofacial bones (either individually or as a composite structure)?

Yes

No

**Q2**

Do you think it is clinically beneficial to assess the dose to **facial bones** for patients who have yet to reach their pubertal growth spurt?

Yes

Maybe

No (optional: state reasoning below)

__________________________________________________

**Q3**

Do you think it is clinically beneficial to assess the dose to **dentition** for patients who have yet to reach their pubertal growth spurt?

Yes

Maybe

No (optional: state reasoning below)

__________________________________________________

**Q4**

To better assess the dose to dentition, the **minor salivary glands** may also need to be considered. Minor salivary glands include lingual (anterior, middle, posterior), buccal, palatal, and labial glands.
 
Does your department routinely delineate the **minor salivary glands** (either separately or as part of the oral cavity)?

Delineated separately

Included within the oral cavity contour

Not considered

**Q5**

In your opinion, what are the main barriers to delineating **dentition or facial bones**, assessing radiation doses or applying dose constraints to these structures in clinical practice routinely?

Time-consuming

Not clinically relevant

No consensus or guidance on what and how to contour dentition and facial bones

Other (please specify below)

__________________________________________________

Display Q6 if ‘Does your department routinely delineate **one or more** dentofacial bones (either individually or as a composite structure)?’ == Yes.

**Q6a**

Please complete the following table on **dentofacial bones** and dose constraints used. Select all that applies and add additional rows to specify **composite** or **other structures** if required.

|  | **Which dentofacial bones are contoured in routine clinical practice?** | **Who contours the dentofacial bones?** | **What radiotherapy dose tolerance is applied/used?** | | | **Optional: What radiotherapy dose tolerance is applied/used?** | |
| --- | --- | --- | --- | --- | --- | --- | --- |
|  | Select all that apply. | Select one from the following drop down. | ALARA (As Low As Reasonably Achievable) | Unknown | Specific dose constraint (specify in the next column). | Specify maximum dose tolerance (Dmax) below. | Specify mean dose tolerance (Dmean)  below. |
| **Mandible** |  | Choose an item. |  |  |  |  |  |
| **Sphenoid bone** |  | Choose an item. |  |  |  |  |  |
| **Orbit** |  | Choose an item. |  |  |  |  |  |
| **Ethmoid bone** |  | Choose an item. |  |  |  |  |  |
| **Maxillary bone** |  | Choose an item. |  |  |  |  |  |
| **Nasal bone** |  | Choose an item. |  |  |  |  |  |
| **Temporomandibular joint (TMJ)** |  | Choose an item. |  |  |  |  |  |
| **Dentition** |  | Choose an item. |  |  |  |  |  |
| **Composite structure (please specify which bones are drawn as one structure)** |  | Choose an item. |  |  |  |  |  |
| **Other (bones, cartilage, minor salivary glands etc.)** |  | Choose an item. |  |  |  |  |  |
| **…** |  |  |  |  |  |  |  |

**Q6b**

Do you use age-adapted dose constraints for **dentition or facial** bones?

Yes (specify for which bones and what constraints are applied)

__________________________________________________

No

Unknown

**Q6c**

If an automated tool is used for contouring of any **dentofacial** bones, please provide more information below (i.e., manufacturer or product name).

________________________________________________________________

**Q6d**

If an automated tool was not used, would you be interested in an automated solution for aiding the delineation of **dentofacial bones**?

Yes

Maybe

No (optional: state reasoning below)

__________________________________________________

**Q6e**

If 'other' was selected as the personnel contouring the **dentofacial** bones, please provide more information below.

________________________________________________________________

**Q6f**

Do you feel confident to contour/review auto-contours for **dentition or facial bones**?

Yes

No

**Q6g**

Would an atlas aid your delineation or review of the autocontours for **dentition or facial bones**?

Yes

No

**Section B Future**

**Q7**

Would you potentially be interested in the following initiatives to investigate dentofacial effects post-radiotherapy for childhood cancer?

Join a working group

Attend an online symposium to discuss current research and/or potential collaboration in this area

Participate in an online contouring workshop for dentofacial bones

I cannot actively participate at this time but I would like to be kept up-to-date on developments via email

None of the above

Other (please specify below)

__________________________________________________

**Q8**

If you are interested in any of the initiatives related to dentofacial effects post-radiotherapy please provide your email address below.

________________________________________________________________

**Q9**

Please provide the name of your department below.

________________________________________________________________
